# A novel gold-nanocluster-based fluorescent sensor for detection of sodium 2-mercaptoethanesulfonate†

**DOI:** 10.1039/c9ra01563a

**Published:** 2019-06-17

**Authors:** Jiaxing Su, Chenchen Feng, Yuan Wu, Jiangong Liang

**Affiliations:** College of Science, Huazhong Agricultural University Wuhan 430070 China yuanwu@mail.hzau.edu.cn +86-27-8728-2133 +86-27-8728-3712

## Abstract

Gold nanoclusters (Au NCs) are widely used in various types of detections due to their unique fluorescence properties. However, there are rare reports on enhanced fluorescence sensors for drug molecules. Here, we report a novel strategy for detection of sodium 2-mercaptoethanesulfonate (MES) by using a fluorescence-enhanced histidine stabilized Au NCs (His-Au NCs) probe. This fluorescence probe showed excellent selectivity and sensitivity towards MES. Furthermore, we have demonstrated that the fluorescence enhancement of His-Au NCs was attributed to ligand exchange with MES by Fourier Transform infrared spectroscopy (FTIR) and X-ray photoelectron spectroscopy (XPS). The feasibility of practical applications of this probe was further investigated by sensing the MES content in Mesna injection (Uromitexan).

## Abstract 2

Schematics illustrating the MES-induced fluorescence enhancement of His-Au NCs for MES quantification because of the surface ligand exchange between His and MES.

## Graphical abstract

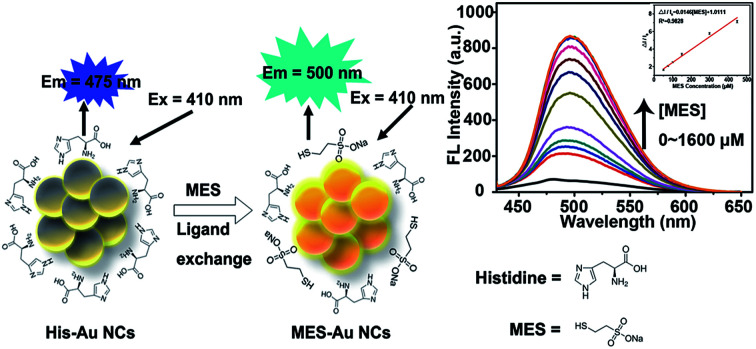

## Introduction

Gold nanoclusters (Au NCs) have attracted extensive attention in the fields of chemistry, chemical biology and functional materials owing to their small size, excellent biocompatibility, highly controllable fluorescence properties, and environmentally friendly synthetic method.^1–12^ Up to now, Au NCs-based fluorescence sensors are mainly dependent on the fluorescence-quenching effect *via* the interactions between Au atoms and the analytes (such as bismerthiazol and Hg^2+^).^13,14^ Previous studies have shown that the fluorescence behavior of Au NCs is not only highly size-dependent, but also correlated with the surface ligands and ligand density.^15^ For example, Zhang *et al.*^16^ successfully prepared Au NC-based fluorescent probes to detect glutathione (GSH) by significantly enhancing the fluorescence intensity of Au NCs, which inspired us to consider the possibility of broadening the applications by developing Au NCs with more functional ligands.

The sodium 2-mercaptoethanesulfonate (MES), a thiol-containing drug, is mainly adopted as an antioxidant in renal protection because of the free thiol group.^17,18^ MES has been reported to be able to scavenge reactive oxygen species (ROS) and improve ischemic acute renal failure.^19^ Moreover, MES was confirmed to be effective in reducing intestinal inflammation in colitis and in preventing hemorrhagic cystitis.^20–22^ With increasing interest in applying MES in treating multifarious disorders, a variety of analytical methods have been developed for detecting MES since 1959.^23^ Most of the currently available detection methods were based on instrumental analysis.^24–27^ For example, Mare *et al.*^24^ achieved MES detection by using chromatography combined with fluorescence detection; Rizk *et al.*^25^ adopted high performance liquid chromatography (HPLC) method for simultaneous separation and quantification of MES in the presence of its degradation products; Salman *et al.*^26^ showed a reliable and specific LC-MS/MS method for detection of MES and Dimesna; Ling *et al.*^27^ performed capillary liquid chromatography to detect MES. Even though the sensitivity of these instrumental analytical methods is relatively high, they are usually complicated in sample pretreatment and high in operation and instrument cost. To address these shortcomings, spectrophotometric methods for the detection of MES were established.^28–31^ Vaishnav *et al.*^28^ developed a simple colorimetric sensor based on the aggregation of spherical Ag NPs caused by MES. Ma *et al.*^29^ and Sroka *et al.*^30^ achieved the quantification of MES by measuring the ultraviolet absorbance of colored substance generated from reaction with MES. Vallvey *et al.*^31^ developed the chemiluminescence (CL) method for MES detection based on the reaction of the MES with Ce(iv). However, these spectrophotometric detection methods required extreme acidic conditions, and most of them were indirect and complicated in operation, suggesting the necessity to develop a fast, simple, and economical method for MES detection.

**Scheme 1:**
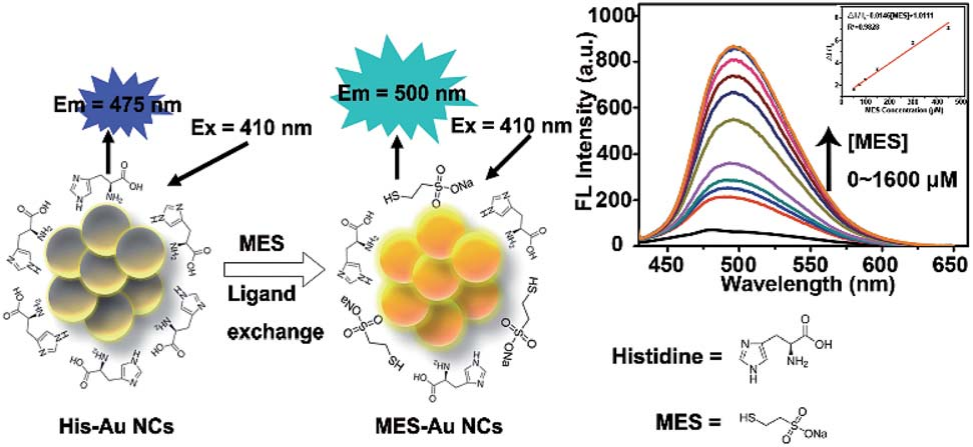
Schematics illustrating the MES-induced fluorescence enhancement of His-Au NCs for MES quantification because of the surface ligand exchange between His and MES.

In our previous work,^32^ the effects of MES-stabilized Au NCs (MES-Au NCs) and histidine-stabilized Au NCs (His-Au NCs) on the propagation of pseudorabies virus were investigated. Occasionally, we found that the fluorescence intensity of His-Au NCs could be enhanced after adding MES. Inspired by this observation, herein we firstly presented novel Au NCs-based fluorescent sensors for detection of MES *via* linear fluorescence enhancement of His-Au NCs in the presence of MES (Scheme 1). The Au NC probes showed high sensitivity and selectivity towards MES even in the presence of disodium 2,2′-dithiobisethane sulfonate (DIMESNA), an inactive dimer of MES formed in the bloodstream. Furthermore, the probes were used to sense the MES content in Mesna injection with satisfactory recovery, suggesting their potential application for MES determination in real samples. Briefly speaking, several merits of this spectrophotometric method based on Au NCs sensors make it attractive for the detection of MES. First, the Au NCs sensors were developed by a simple, environmentally friendly synthetic method with minimizing cost; second, this spectrophotometric method is based on the fluorescence enhancement of Au NCs directly caused by MES, and hence highly sensitive to MES. Third, we have found that the reason for fluorescence enhancement of Au NCs is the formation of new Au–S surface ligands, which would extend the research on the ligand's role in the fluorescence of gold nanoclusters and widen the application of Au NCs in sensing based on ligand exchange principle.

## Results and discussion

His-Au NCs and MES-Au NCs were synthesized as previously reported method.^32,33^ The high-resolution transmission electron microscopy (HR-TEM) analysis showed no obvious difference between His-Au NCs and MES-Au NCs in morphology (Fig. S1a and b†). The sizes of His-Au NCs and MES-Au NCs were about 1.2 and 2.2 nm, respectively.

**Fig. 1:**
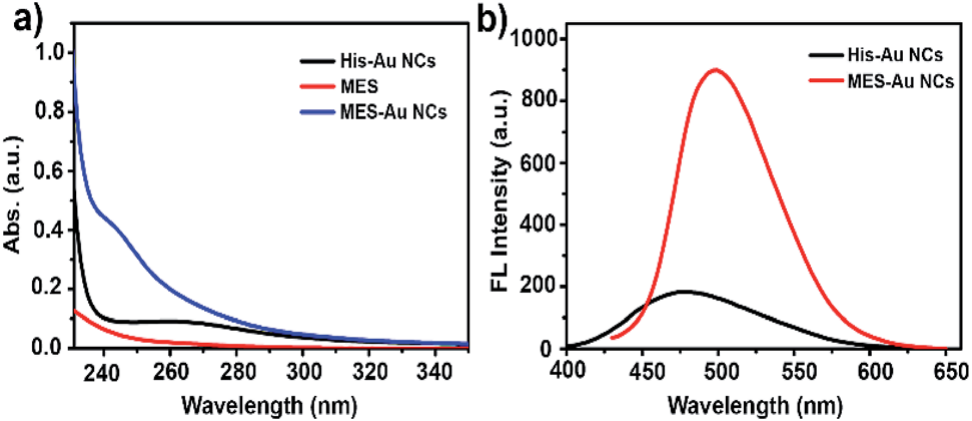
(a) The UV-Vis absorption spectra of His Au NCs, MES, and MES-capped Au NCs; (b) the fluorescence emission spectra of His Au NCs and MES-capped Au NCs under the excitation of 410 nm.

Fig. 1a and b show the ultraviolet-visible (UV-vis) absorption and fluorescence spectra (FL) of His-Au NCs and MES-Au NCs (MES : Au = 24 : 1). The fluorescent spectra of MES-Au NCs showed a maximum emission peak centered at 500 nm, an obvious red shift from that of His-Au NCs (475 nm) under the excitation of 410 nm. Additionally, the fluorescence of Au NCs had ten-fold enhancement after adding MES into His-Au NCs. The quantum yield of MES-Au NCs (MES : Au = 24 : 1) was estimated to be about 4.33% by using quinine sulfate as a reference in contrast to that of about 1.08% for His-Au NCs.

To further explore the fluorescence enhancing mechanism, both His- and MES-capped gold clusters were characterized by Fourier Transform infrared spectroscopy (FTIR) and X-ray photoelectron spectroscopy (XPS). Firstly, the capping ligands on the surfaces of Au NCs were characterized by FTIR (Fig. S3†). A peak at 2571 cm^−1^ attributed to the S–H stretching vibration can be observed in the spectrum of MES, but this characteristic peak is absent in the spectrum of MES-Au NCs. Both the spectra of MES-Au NCs and MES showed the stretching vibrations of O=S=O at 1251 and 1179 cm^−1^, strongly suggesting that MES anchors on the surface of Au NCs through the sulfur atom and Au atom by the formation of Au–S bond.^34,35^ Meanwhile, both gold clusters exhibited the characteristic IR bands of imidazole ring (imidazole stretches; 1464 cm^−1^) of histidine,^36^ confirming the partial replacement of His-ligands on the surfaces of His-Au NCs by MES. The ligand exchange could be further verified by X-ray photoelectron spectroscopy (XPS). Specifically, the XPS spectra of His-Au NCs (Fig. S4a†), MES-Au NCs (Fig. S4b†) and the high resolution spectra of S (2p) (Fig. S4d†) showed that the peak of S element offered by MES only appeared in MES-Au NCs, indicating the generation of Au–S bond in MES-Au NCs.

**Fig. 2:**
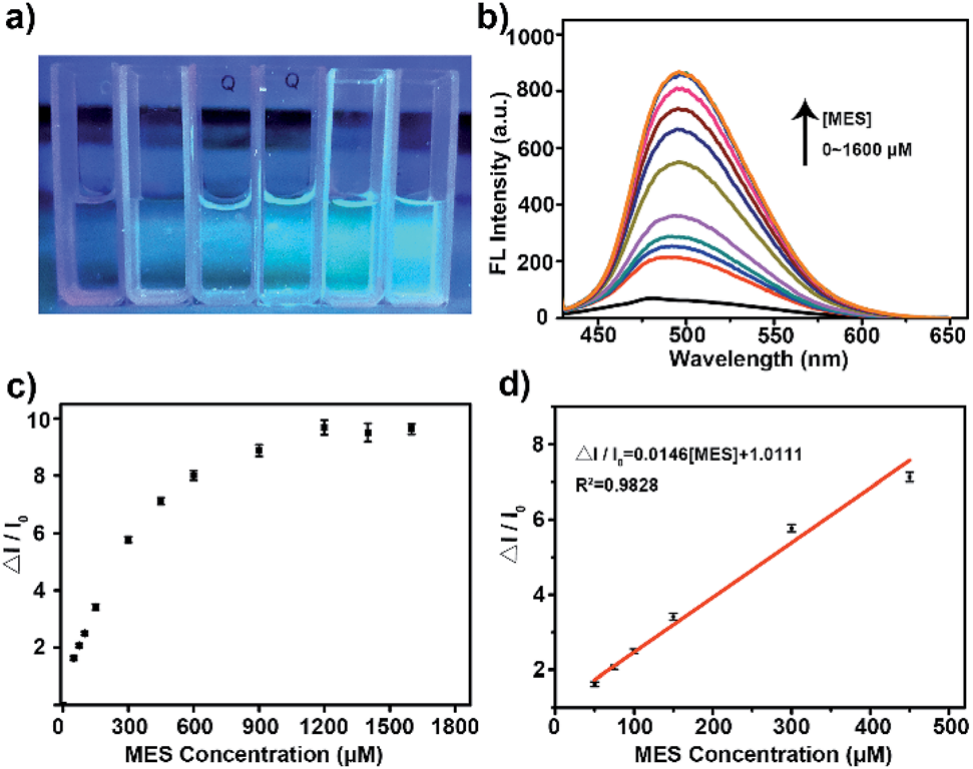
(a) From left to right: the photos of His-Au NCs (50 μM) with different concentrations of MES (0, 50, 200, 600, 1200 μM); (b) fluorescence response of His-Au NCs (50 μM) with different concentrations (50–1600 μM) of MES under 410 nm irradiation; (c) plot of the change between Δ*I*/*I*_0_ and different MES concentrations (50–1600 μM); (d) plot of the relationship between Δ*I*/*I*_0_ and different MES concentrations (50–450 μM).

Then the potential application of the His-Au NCs probes in the detection of MES was tested. As shown in Fig. 2a, the addition of MES significantly increased the fluorescence of His-Au NCs. A ten-fold fluorescence enhancement of His-Au NCs by adding different concentration of MES was observed in the fluorescence spectra (Fig. 2b and c). Fig. 2d showed the variation of fluorescence intensity (Δ*I*/*I*_0_) at 500 nm as a function of MES concentration ([MES]). The dependence of Δ*I*/*I*_0_ on MES concentration followed the equation: Δ*I*/*I*_0_ = 0.01458[MES] + 1.01114 (MES: μM, *R*^2^ = 0.984) from MES concentration of 50 to 450 μM. The limit of detection (LOD) for MES was 4.5 μM at a signal-to-noise ratio of 3. Table S1† shows the comparison of our MES detection method with previously reported ones. It can be seen that although not the lowest in detection limit, our method is relatively simple, economical and direct when compared with other indirect method or the method requiring acidic condition.

**Fig. 3:**
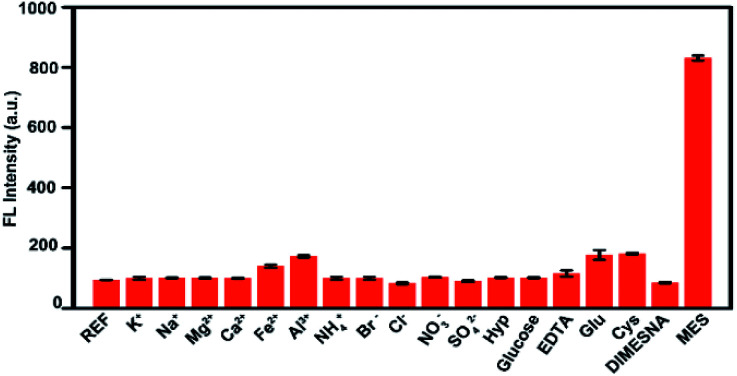
Fluorescence responses of His-Au NCs (50 μM) with different interferences (600 μM) and MES (600 μM).

Furthermore, the specificity and selectivity of His-Au NCs probes were tested by several control experiments using K^+^, Na^+^, Mg^2+^, Ca^2+^, Fe^2+^, Al^3+^, NH_4_^+^, Cl^−^, Br^−^, SO_4_^2−^, NO_3_^−^, hydroxyproline (Hyp), glutamic (Glu), cysteine (Cys), EDTA·Na_2_ and DIMESNA as interferences. As shown in Fig. 3, it can be seen that the fluorescence intensity of His-Au NCs was slightly increased by glutamic (Glu) and cysteine (Cys), but not significantly affected by the other interferences, indicating that His-Au NCs could sensitively distinguish MES from DIMESNA and other widely available interferences.

Table S2† shows the sensitivity of the His-Au NCs to the content of MES in the Mesna injection (Uromitexan) by standard addition method. It can be seen that the relative standard deviation was less than 3.4%, and the recovery was between 97.1% and 104.6%, indicating that His-Au NCs could be potentially used as probes for quantitative detection of MES in Mesna injection.

## Conclusion

Our work presented a simple, economical, sensitive, and selective method for detecting MES by fluorescence-enhanced probes based on His-Au NCs. MES was found to enhance the fluorescence of His-Au NCs by replacing the His-ligands through the formation of Au–S bond, leading to linear enhancement of fluorescence intensity in response to MES over the range of 50 to 450 μM with high sensitivity (LOD = 4.5 μM, S/N = 3). To our best knowledge, this is the first report about the direct spectrometry method for MES detection using His-Au NCs probes under relatively mild condition. The His-Au NCs probes show excellent selectivity towards MES in the presence of most common interferences. Moreover, the His-Au NCs probes exhibit satisfactory recovery when used to sense the MES content in Mesna Injection, suggesting their potential application for MES determination in real samples.

## Experimental section

### Reagents

Sodium 2-mercaptoethanesulfonate (MES), l-histidine (His) and glucose (glucose) were purchased from Aladdin Reagent Co., Ltd. disodium (DIMESNA) was acquired from ApexBio Reagent Co., Ltd. Cysteine (Cys) and chloroauric acid (HAuCl_4_) were obtained from Sigma-Aldrich Reagent Co., Ltd. Potassium chloride (KCl), sodium chloride (NaCl), magnesium chloride hexahydrate (MgCl_2_·6H_2_O), calcium chloride dihydrate (CaCl_2_·2H_2_O), aluminum chloride hexahydrate (AlCl_3_·6H_2_O), potassium bromide (KBr), ammonium chloride (NH_4_Cl), iron sulfate heptahydrate (FeSO_4_·7H_2_O), sodium sulfate (Na_2_SO_4_), sodium nitrate (NaNO_3_), ethylenediaminetetraacetic acid disodium salt (EDTA·Na_2_), glutamic acid (Glu) and proline (Hyp) were acquired from Sinopharm Chemical Reagent Co., Ltd. Mesna injection (Uromitexan) was obtained from Qilu Pharmaceutical (Hainan) Co., Ltd. The deionized water was used in this study. All chemicals and solvents were of analytical grade or better and used without further purification.

### Apparatus

The UV-vis absorption spectra were recorded on a UV-2450 spectrophotometer (Shimadzu, Japan). X-ray photoelectron spectroscopy (XPS) was recorded on a VG Multilab 2000 X-ray photoelectron spectrometer (Thermo VG, UK). Fluorescence measurements were performed using an RF-5301 PC spectrofluorometer (Shimadzu, Japan). Fourier transform infrared spectra (FTIR) were obtained to identify the molecular structures of Au NCs with a Nicolet Avatar-330 spectrometer (Thermo Nicolet, USA) through the KBr pellet technique. The size and morphology of Au NCs were recorded on a HRTEM JEM-2100F instrument (JEOL, Japan). All deionized water was obtained from a Milli-Q ultrapure water machine (Millipore, USA).

### Preparation of (a) His-Au NCs and (b) MES-Au NCs

(a) Au nanoclusters were synthesized by blending histidine and chloroauric acid as reported previously.^25,26^ Briefly, chloroauric acid (5 mL, 0.01 M) was added to the solution of histidine (15 mL, 0.1 M), then stored at 25 °C for 2 h without light. The concentration of the as-synthesized His-Au NCs was 2500 μM.

(b) His-Au NCs (10 mL, 2500 μM) was mixed with MES (10 mL, 60 mM), and maintained for reaction overnight without light.

### Interaction experiment between His-Au NCs and MES

A solution of MES at a concentration of 0.01 M was separately diluted with deionized water to obtain 250, 375, 500, 750, 1500, 2250, 3000, 4500, 6000, 7000, and 8000 μM MES solutions. An aqueous solution of histidine (200 μL, 250 μM) was added to 200 μL of different concentrations of MES solution. The final volume was 1 mL. The fluorescence intensity was measured for each set of samples after reacting for overnight 25 °C in the dark. Each set of experiments was repeated three times.

### Selective experiment

The solutions of KCl, NaCl, MgCl_2_, CaCl_2_, EDTA·Na_2_, Glu, Cys, AlCl_3_·6H_2_O, KBr, NH_4_Cl, FeSO_4_·7H_2_O, Na_2_SO_4_, NaNO_3_, DIMESNA and MES were separately prepared at a concentration of 3000 μM. Then, 200 μL of each solution was mixed with His-Au NCs solution (200 μL, 250 μM) with a final volume of 1 mL. The fluorescence intensity was measured for each set of samples after reacting for overnight 25 °C in the dark. Each set of experiments was repeated three times.

### Detection in actual samples

(a) The Uromitexan solution (200 μL, 2000 μM) was mixed with His-Au NCs solution (200 μL, 250 μM) at a volume of 1 mL with deionized water.

(b) Two sets of the Uromitexan solution (200 μL and 2000 μM) were mixed with MES standard solutions (600, 800, and 1000 μM) at a final volume of 1 mL, respectively. Then, the obtained solutions (500 μL) were separately added to His-Au NCs solution (200 μL, 250 μM) at final volume of 1 mL with deionized water. After reaction overnight at 25 °C in the dark, the fluorescence intensity was measured for each set of samples. Each set of experiments was repeated three times.

## Conflicts of interest

There are no conflicts of interest to declare.

## Supplementary Material

RA-009-C9RA01563A-s001
