# Supplementary material for: A novel gold-nanocluster-based fluorescent sensor for detection of sodium 2-mercaptoethanesulfonate
Source: RSC Adv. 2019 Jun 17;9(33):18949–53. doi: 10.1039/c9ra01563a (PMC9065114; doi:10.1039/c9ra01563a)
Supplement: RA-009-C9RA01563A-s001 [file RA-009-C9RA01563A-s001.pdf]

Electronic Supplementary Material (ESI) for RSC Advances.
This journal is © The Royal Society of Chemistry 2019

Supporting Information

A novel gold-nanocluster-based fluorescent sensor for detection of sodium 2-mercaptoethanesulfonate

Jiaxing Su‡, Chenchen Feng‡, Yuan Wu and Jiangong Liang*

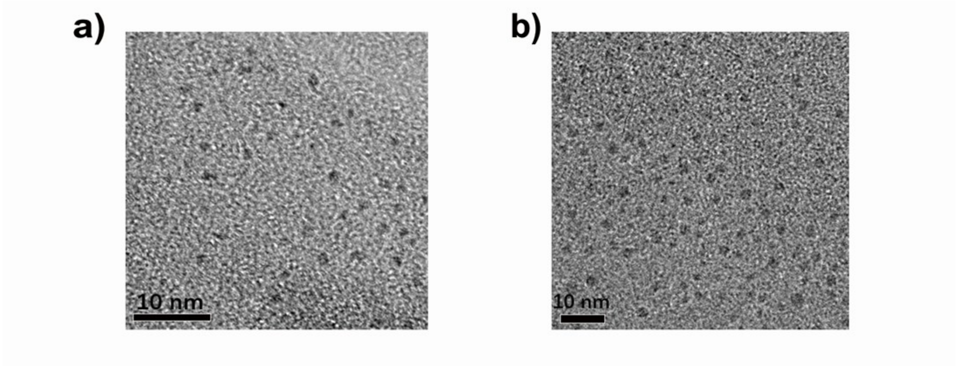

Fig. S1. The HR-TEM images of (a) His-Au NCs and (b) MES-Au NCs.

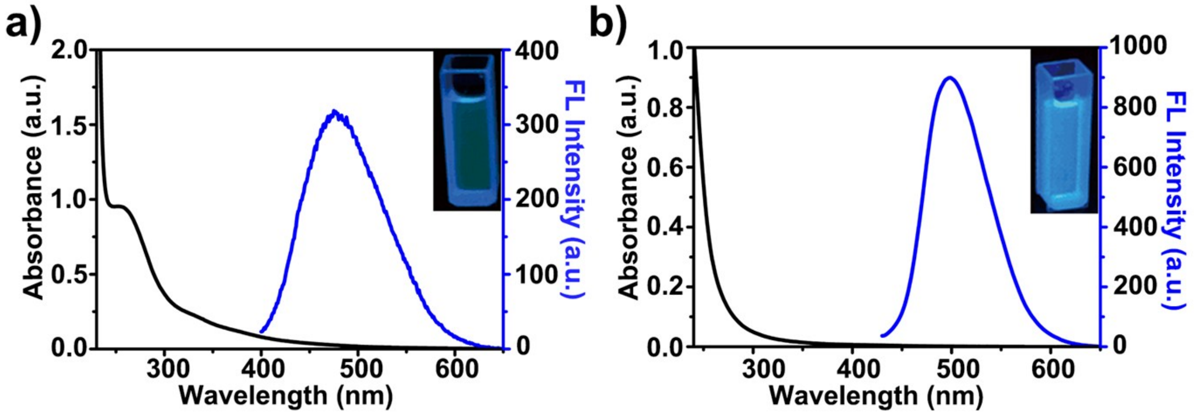

Fig. S2. UV absorption spectra (black line) and fluorescence emission spectra (blue line) under the excitation of 410 nm of (a) His-Au NCs and (b) MES-Au NCs.

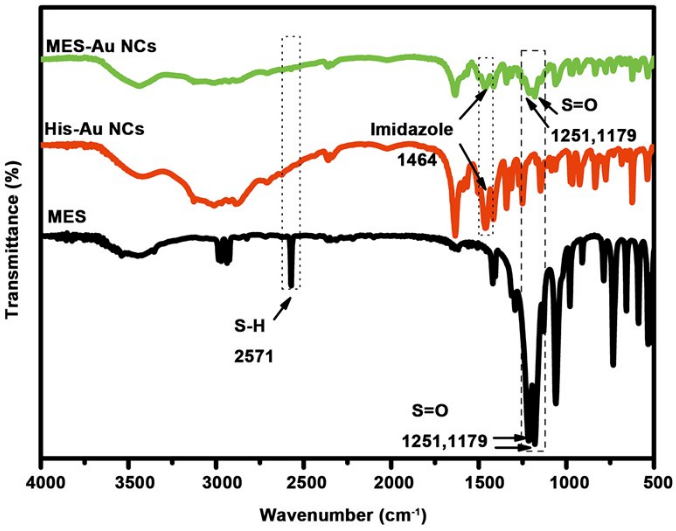

Fig. S3. The FT-IR data of His-Au NCs, MES and MES-capped Au NCs.

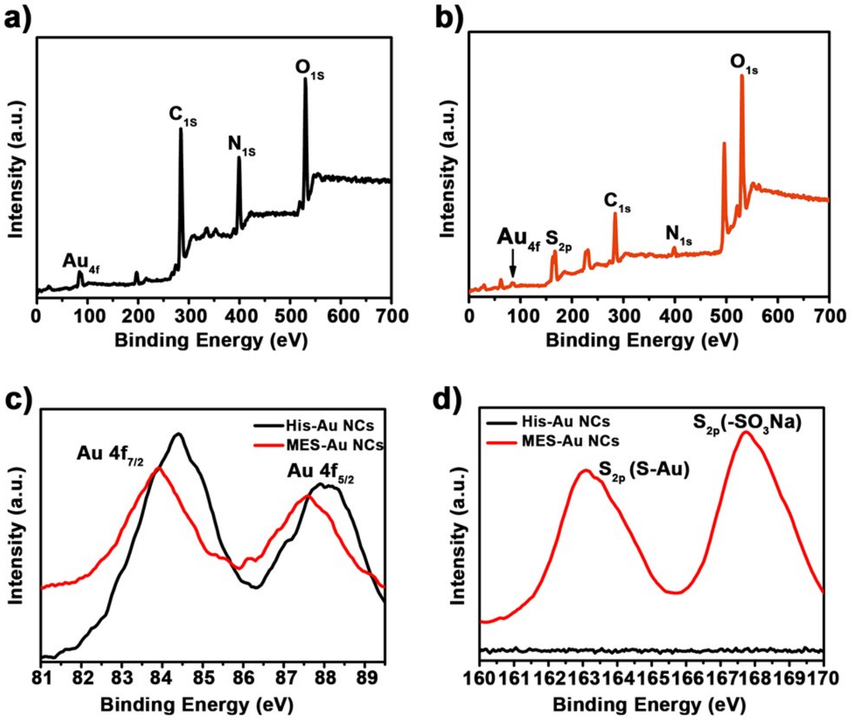

Fig. S4. XPS spectra of (a) Survey spectrum of His-Au NCs; (b) Survey spectrum of MES-Au NCs; (c) S2p region of His-Au NCs and MES-Au NCs, respectively; (d) Au_{4f} region of His-Au NCs and MES-Au NCs, respectively.

Table S1 Comparison of analytical methods for MES detection

No.	Method	LOD（Limit of detection）	Reference
1	Reverse phase HPLC	1.64 nM per 20 μL injection volume	Biomed. Chromatogr., 2005, 19, 80-86.
2	HPLC	7.5 μg/ml	J. Chromatogr. Sci., 2015, 53, 742-748.
3	LC-MS/MS	0.24 PPB	Expert Rev. Anticancer Ther., 2016, 16, 123-130.
4	Spectrophotometric method (leucoc rystal violet)	0.10 μg/mL	J. Anal. Chem., 2015, 70, 831-836.
5	Spectrophotometric method (Ferric solution)	1.9 μM	Asian J. Chem., 2011, 23, 3522-3524.
6	Spectrophotometric method (Ag NPs)	10.2 μM	Spectrochim. Acta, Part A, 2017, 179, 155-162.
7	Chemiluminescence (Cerium, IV)	8.4 μM	Talanta, 2000, 51, 1155-1161.
8	Spectrophotometric method (Au NCs)	4.5 μM	This work

Table S2 Determination of MES in uromitexan by the standard addition method.

Nominal concentration (μM)	MES detected amount (μM)	The relative standard deviation (%)	MES added amount (μM)	Total detected amount (μM)	Recovery rate (%)
400.0	377.1	3.4	200	583.2	103.1
400.0	379.7	2.3	400	778.66	97.1
400.0	380.5	1.8	600	1008.0	104.6

References

1 S. Mare, S. Penugonda and N. Ercal, *Biomed. Chromatogr.*, 2005, **19**, 80-86.
2 M. Rizk, E. A. Taha, S. Mowaka and Y. M. Abdallah, *J Chromatogr Sci*, 2015, **53**, 742-748.
3 D. Salman, J. Swinden, J. M. Peron, S. Barton and S. Nabhani-Gebara, *Expert Rev Anticancer Ther*, 2016, **16**, 123-130.
4 S. K. Vaishnav, K. Patel, K. Chandraker, J. Korram, R. Nagwanshi, K. K. Ghosh and M. L. Satnami, *Spectrochim. Acta, Part A*, 2017, **179**, 155-162.
5 W. X. Ma, N. Li, O. Sha and Y. H. Liu, *Asian J. Chem.*, 2011, **23**, 3522-3524.
6 B. Szpikowska-Sroka and A. Guz, *J. Anal. Chem*., 2015, **70**, 831-836.
7 L. F. Capitán-Vallvey, M. C. V. Miron and R. A. Acosta, *Talanta*, 2000, **51**, 1155-1161.
